# Sanjad–Sakati syndrome: integrated emergency care, long-term management, and expert perspectives—a narrative review

**DOI:** 10.3389/fped.2026.1761285

**Published:** 2026-07-14

**Authors:** Meshal Atiyah

**Affiliations:** Pediatric Emergency Consultant, Emergency Department, Security Forces Hospital, Makkah, Saudi Arabia

**Keywords:** endocrine crisis, genetic disorders, hypocalcemia, hypoparathyroidism, pediatric emergency, Sanjad–Sakati syndrome, TBCE mutation

## Abstract

**Background:**

Sanjad–Sakati syndrome (SSS) is a rare autosomal recessive disorder characterized by congenital hypoparathyroidism, persistent hypocalcemia, growth retardation, and distinctive craniofacial dysmorphism. Despite its rarity, SSS is more prevalent in the Middle East due to high consanguinity rates. Its multisystem involvement—including endocrine, neurologic, respiratory, renal, dental, and immunologic systems—poses significant challenges in pediatric emergency care and long-term management.

**Objective:**

This review aims to provide a comprehensive, clinically focused overview and expert opinion on the emergency management, diagnostic evaluation, and acute and long-term treatment strategies for SSS, with an emphasis on high-risk manifestations such as hypocalcemic crises, airway instability, immune vulnerability, and difficult vascular access.

**Findings:**

Children with SSS often present with hypocalcemia, seizures, tetany, apnea, or laryngospasm, necessitating urgent calcium correction and airway stabilization. Microtubule dysfunction due to tubulin-specific chaperone E gene mutations contributes to endocrine and structural abnormalities, leading to neurodevelopmental impairment, sleep-disordered breathing, nephrocalcinosis, dental anomalies, and heightened susceptibility to infections associated with immune deficiency. Emergency care is complicated by abnormal airway anatomy, challenging intravenous access, susceptibility to sepsis, and metabolic instability. Early identification of biochemical abnormalities remains the cornerstone of diagnosis and timely stabilization. Long-term care requires lifelong calcium and vitamin D supplementation, regular metabolic monitoring, renal assessment, psychosocial support, and caregiver education.

**Conclusion:**

SSS demands clinical vigilance due to its multisystem involvement and potential for life-threatening metabolic and respiratory events. Early biochemical correction, cautious airway management, infection prevention, and coordinated multidisciplinary follow-up are critical to improving outcomes and reducing morbidity and mortality. Implementing standardized emergency protocols, family-directed action plans, and genetic counseling in high-risk populations can significantly enhance long-term prognosis for affected children.

## 1 Introduction

Sanjad–Sakati syndrome (SSS), also known as hypoparathyroidism–retardation–dysmorphism (HRD) syndrome, is a severe autosomal recessive TBCE-related disorder. It has historically been discussed alongside, and in some literature classified within, the autosomal recessive Kenny–Caffey syndrome type 1 (KCS1) spectrum. Although these entities substantially overlap, the recessive Kenny–Caffey phenotype may show more prominent skeletal abnormalities, including cortical thickening and medullary stenosis. SSS is characterized by infantile-onset hypoparathyroidism with marked hypocalcemia, prenatal and postnatal growth failure, distinctive craniofacial dysmorphism, developmental delay, and intellectual disability (1, 2). Affected infants typically present during the neonatal or early infancy period with hypocalcemic tetany or seizures resulting from parathyroid hormone (PTH) deficiency (3). Orofacial manifestations are highly valuable in distinguishing SSS from other causes of neonatal hypocalcemia and often include deep-set eyes, a narrow face, micrognathia, a beaked nose, thin lips, and small hands and feet (4, 5).

## 2 Epidemiology and genetic basis

Although the global burden of SSS remains indeterminate because of case scarcity, misdiagnosis, and underreporting, data from the Arabian Peninsula indicate an estimated incidence of approximately 1 per 100,000 live births in Saudi Arabia, with a comparable prevalence in other Gulf Cooperation Council countries (6, 7). Rare case reports have also been documented outside the Arabian Peninsula (8–13). SSS occurs in offspring carrying pathogenic variants in the tubulin-specific chaperone E (TBCE) gene located on chromosome 1q42.3, most likely due to the high rate of consanguinity among Middle Eastern populations, particularly in the Arabian Peninsula (14). The TBCE gene encodes a molecular chaperone involved in the folding of *α*-tubulin and *β*-tubulin monomers and the assembly of *α*/*β*-tubulin heterodimers, which are essential components of microtubule formation, contributing to cell division, motility, and intracellular transport (15, 16). Mutations in the TBCE gene lead to defective production of the chaperone protein, resulting in abnormal hydrophobic interactions between tubulins and other cellular proteins. These aberrant interactions prevent the proper construction of functional quaternary structures, as evidenced by reduced microtubule density at the microtubule-organizing center, altered microtubule polarity, and disruption of Golgi and late endosomal compartment ultrastructure (17). Because tubulin proteins are expressed in all body cells, this underlies the multisystem manifestations observed in SSS (15, 18). Recent genotype–phenotype analyses indicate that biallelic TBCE variants produce a broader phenotypic spectrum than classic HRD/SSS alone. In a combined series and literature review of 335 individuals with TBCE-related disorders, SSS represented the predominant phenotype (85%), whereas childhood-onset neurodegenerative phenotypes accounted for approximately 5%. These rarer disorders may feature progressive spastic–ataxic tetraparesis, optic atrophy, distal motor axonal neuropathy, and progressive corpus callosum or cerebellar abnormalities. Recognition of this broader TBCE spectrum is particularly relevant when neurological deterioration appears progressive or disproportionate to hypocalcemia alone (19).

## 3 Clinical presentation and systemic complications

### 3.1 Endocrinological manifestations

The hallmark manifestation of SSS is hypocalcemic tetany or seizures, frequently accompanied by irritability, apnea, or laryngospasm caused by severe hypocalcemia resulting from low circulating PTH. This deficiency is likely due to impaired microtubule-dependent intracellular trafficking and disrupted organogenesis of the developing parathyroid glands, leading to congenital hypoparathyroidism (20, 21). In this context, Anteet et al. reported a high incidence of thyroid dysfunction in patients with SSS due to subclinical autoimmune hypothyroidism (Hashimoto's thyroiditis), evidenced by the presence of thyroid autoantibodies in a significant number of cases, emphasizing the need for routine thyroid screening in this population (22). In addition, abnormalities in other endocrine axes may occur, including growth hormone (GH) deficiency and altered pituitary-gonadal function, likely reflecting the broader effects of microtubule dysfunction on endocrine tissues (23, 24). Growth retardation is a common pre- and postnatal feature among most affected infants. This growth deficit, combined with craniofacial dysmorphism—including a narrow face, deep-set eyes, a beaked or depressed nasal bridge, micrognathia, a thin upper lip, large floppy ears, and small hands and feet—is a highly recognizable feature of SSS (13, 25). Hafez et al. identified GH deficiency in patients with SSS that responded poorly to standard stimulation tests with arginine and L-DOPA; however, a partial response was observed with clonidine and notable improvement in both height and weight after treatment with replacement human GH (13). Furthermore, the inherent microtubular defect impairs chondrocyte proliferation and maturation at the growth plates, thereby restricting longitudinal bone growth (24). More recent cohort data indicate that the endocrine phenotype of HRD syndrome extends beyond congenital hypoparathyroidism. In a retrospective cohort of 58 patients who underwent endocrine evaluation, hypothyroidism was identified in 36%, adrenal glucocorticoid insufficiency in 22% of those tested, and growth hormone deficiency in 28% of those investigated; symptomatic hypoglycemia resulted in hospitalization in 55% of patients. These findings support periodic thyroid and adrenal screening, together with a low threshold to evaluate hypoglycemia and additional pituitary dysfunction in affected children (26).

### 3.2 Neurological manifestations

Multiple interrelated factors likely contribute to the neurological manifestations of SSS, including the effects of early-life hypocalcemia, structural neurodevelopmental abnormalities resulting from defective microtubule and chaperone pathways, and secondary intracranial calcifications. Neurological symptoms such as recurrent seizures, microcephaly, and developmental delay may persist beyond the acute hypocalcemic episode due to structural brain injury or basal ganglia calcifications, which have been radiologically confirmed in nearly one-third of affected children (3, 27). Elhassanien et al. reported a case series of 24 patients with SSS, in which a history of consanguinity was identified in 75% of cases. All patients exhibited features including microcephaly, developmental delay, intellectual disability, and seizures. Computed tomography (CT) and magnetic resonance imaging (MRI) revealed intracranial calcifications in 29.2% of the cohort. Additional structural abnormalities included a thin corpus callosum in two patients and intraventricular hemorrhage in one patient (3). Neuroimaging studies of individuals with SSS have also demonstrated reduced white matter volume in approximately 30% of one cohort (28), while structural anomalies such as partial agenesis of the corpus callosum have been reported rarely (29). Although uncommon, severe neurological events such as status epilepticus have been observed in this population (27).

### 3.3 Dental and orofacial anomalies

Dental abnormalities such as enamel defects, small dental arches, enamel hypoplasia, oligodontia, microdontia, delayed eruption, taurodontism, and dental caries are frequently reported as direct consequences of developmental hypocalcemia and underlying ectodermal dysplasia–like features (30–32). A systematic review that included 56 case reports of patients with SSS identified enamel hypoplasia, hypodontia, microdontia, small dental arches, high-arched palate, deep overbite, and increased overjet as common dental deformities (4). Prenatal hypocalcemia and hyperphosphatemia due to congenital PTH deficiency may impair amelogenesis and tooth development, leading to enamel hypoplasia and root maturation failure (30).

### 3.4 Ophthalmologic manifestations

Ophthalmologic findings—including corneal opacity, microphthalmia, nanophthalmos, and tortuous retinal vessels—have been reported in multiple case studies, possibly resulting from defective microtubule organization and calcium deposition in ocular tissues (33–35). In a study of 17 children with SSS, all participants exhibited microphthalmia and retinal vascular tortuosity on funduscopic examination, while 47% had esotropia, 23% had exotropia, and 94% showed significant hyperopic astigmatism (33). Anterior segment findings frequently described include microphthalmia or nanophthalmos and reduced corneal diameters in earlier reports (36).

### 3.5 Otolaryngologic features

Children with SSS commonly present with distinctive otolaryngologic and airway-related abnormalities reflecting craniofacial dysmorphism, skeletal anomalies, respiratory compromise, and susceptibility to recurrent infections (37, 38). Laryngoscopic examination in these patients often reveals multiple anomalies, including a retroflexed epiglottis, markedly redundant supraglottic mucosa, and limited airway exposure. These features likely contribute to upper airway narrowing, altered airway configuration, and increased risk of obstructive events, which may complicate tracheostomy or endotracheal intubation in this patient population (39).

### 3.6 Respiratory complications

Due to craniofacial dysmorphism, which increases the risk of airway obstruction and aspiration, children with SSS are prone to multiple respiratory complications, ranging from recurrent respiratory infections and airway abnormalities to sleep-disordered breathing (SDB), central hypoventilation, pulmonary hypertension, and respiratory failure (40, 41). The development of SDB in SSS results from a combination of anatomical and neurological factors. Features such as micrognathia, a restrictive thoracic cage, and growth failure may predispose affected individuals to upper airway obstruction and decreased ventilatory reserve, leading to hypoventilation. In addition, microtubule dysfunction affecting the central respiratory centers impairs ventilatory control, resulting in central apneas and inadequate respiratory effort. A case series of 12 genetically confirmed Omani patients with SSS aged 2–17 years reported obstructive sleep apnea in all cases, with central apnea and sleep-related hypoventilation present in 33%. Eight patients experienced severe SDB. Two developed pulmonary hypertension secondary to SDB and subsequently died from type II respiratory failure (40). Another report described two female siblings (aged 14 and 19 years) with SSS who presented with daytime fatigue, tachypnea, and oxygen desaturation. Physical examination revealed congenitally small oral and thoracic cavities, and polysomnographic evaluation demonstrated central sleep apnea and significant hypoventilation that worsened during rapid eye movement (REM) sleep (42).

### 3.7 Renal complications

Children with SSS are at increased risk of nephrocalcinosis, nephrolithiasis, and progressive renal impairment arising from a combination of therapy-induced hypercalciuria, PTH deficiency–related disturbances in renal tubular calcium and phosphate handling, and potential intrinsic renal vulnerability associated with microtubule and chaperone dysfunction. In a case series of 17 patients with a median age of 10.7 years, 59% developed bilateral medullary nephrocalcinosis, and one progressed to end-stage renal disease (38). Another report described an 11-year-old female with SSS who, despite having no history of urinary tract infection, developed a 12-mm staghorn calcium phosphate renal stone along with medullary nephrocalcinosis (43). The absence of normal PTH signaling reduces the kidney's ability to reabsorb calcium in the distal tubules and excrete phosphate, leading to persistently elevated serum phosphate and low calcium levels. Under conditions of low PTH and impaired feedback regulation, chronic administration of calcium and vitamin D for the correction of hypocalcemia increases the filtered calcium load, predisposing to hypercalciuria and calcium deposition within the renal medulla. Furthermore, the TBCE mutation–related tubulin-folding defect may compromise renal tubular cell architecture, heighten susceptibility to injury, and promote interstitial calcification.

## 4 Assessment and diagnosis

### 4.1 Laboratory biomarkers

Affected infants typically present with hypocalcemia related to low PTH levels and elevated serum phosphate, most often showing total serum calcium concentrations below the normal range (<8.8 mg/dL) and phosphate concentrations between 4.0 and 7.0 mg/dL (20). Measurement of intact PTH is essential in the presence of hypocalcemia to confirm true hypoparathyroidism rather than secondary hypocalcemia. Aminzadeh and colleagues reported a series of 29 genetically confirmed cases of SSS. PTH values available for nearly half of the patients ranged from <0.4 to 7.5 pg/mL, serum calcium ranged from 5 to 7 mg/dL, and serum phosphorus ranged from 6.4 to 13 mg/dL. Alkaline phosphatase (ALP) levels were normal for age (<700 U/L) in all patients except one (1,350 U/L), who had a markedly low serum vitamin D concentration (44). Another case report described a 4-month-old female with hypoparathyroidism-associated hypocalcemia, presenting with a serum calcium concentration of 39 mg/L, serum phosphate of 75 mg/L, magnesium of 17 mg/L, PTH of 5.5 pg/mL, vitamin D of 7.7 ng/mL, ALP of 235 IU/L, and a urinary calcium/creatinine ratio of 0.20 mmol/L (20). Similarly, Bali et al. reported an SSS newborn with comparable biomarker patterns in PTH, calcium, phosphate, and vitamin D levels (45). Recognizing the characteristic triad of low calcium, high phosphate, and low PTH in an infant exhibiting the clinical and morphological features of SSS is critical for prompt diagnosis and management.

### 4.2 Imaging

Cranial CT and MRI in children with SSS often reveal intracranial calcifications, particularly in the basal ganglia, globus pallidus, or internal capsule. In one cohort of 24 patients, cranial CT/MRI demonstrated calcifications in 29% of those examined (3). MRI and CT also identify structural brain abnormalities in individuals with SSS, most notably hypoplasia of the anterior pituitary gland and corpus callosum, as well as reduced white matter volume reported in approximately 30% of affected individuals (28). Evaluation of the growth hormone axis, thyroid and adrenal function, neurodevelopmental status, and electroencephalography (EEG) in cases of seizures can assist in assessing the growth failure, developmental delay, and neurocognitive impairment characteristic of SSS. In addition, polysomnography and EEG are recommended for children with SSS presenting with SDB, suspected hypoventilation, or pulmonary hypertension.

Given the increased risk of nephrocalcinosis, nephrolithiasis, and renal tubular abnormalities, renal ultrasonography should be included in the routine diagnostic workup of patients with SSS. Elhassanien et al. reported renal calcifications in 16 of 24 patients on ultrasound (3). Skeletal radiographs in the same cohort demonstrated delayed bone age and medullary stenosis in 91.7% and 8.3% of cases, respectively (3). Another case report described an 11-year-old female with SSS who had a 12-mm staghorn calcium phosphate renal calculus identified on CT (43). In the context of respiratory complications, chest imaging may demonstrate thoracic skeletal deformities and a small thoracic cavity.

### 4.3 Genetic workup

Genetic confirmation of SSS is achieved by identifying pathogenic variants in the TBCE gene, particularly in infants or children presenting with the characteristic triad of congenital hypoparathyroidism with hypocalcemia and hyperphosphatemia, prenatal or postnatal growth failure, and distinctive facial dysmorphism. Prenatal genetic testing is also recommended in high-risk families with a history of consanguinity or previously affected offspring (12).

The primary gene implicated in SSS is the TBCE gene, located on chromosome 1q42–43, which commonly harbors a founder 12–base pair deletion in exon 3 (c.155_166del; p.Ser52_Gly55del). Although most molecularly confirmed patients carry this deletion, rare cases lacking TBCE mutations have been reported, suggesting possible genetic heterogeneity (46).

## 5 Diagnostic algorithm for SSS

### 5.1 Clinical suspicion in the emergency department or early pediatric presentation

When SSS is suspected, perform immediate evaluation of serum calcium, ionized calcium, phosphate, magnesium, and PTH levels, along with concurrent assessment of blood glucose and electrolytes to exclude additional metabolic disturbances. If the patient presents with any of the following features, prompt investigation for SSS is warranted:

▪ Neonatal or early-infantile hypocalcemic seizures, tetany, or carpopedal spasms.
▪ Low birth weight or failure to thrive
▪ Dysmorphic facial features (microcephaly, deep-set eyes, beaked nose, thin upper lip, micrognathia)
▪ Short stature or postnatal growth retardation
▪ Developmental delay (motor or cognitive)
▪ Family history of consanguinity or similar phenotypic presentation
▪ Immediate Emergency Evaluation:
▪ Urgently measure serum total and ionized calcium.
▪ Measure serum phosphate, magnesium, and PTH concentrations.
▪ Assess blood glucose and serum electrolytes to rule out concurrent metabolic abnormalities.

### 5.2 Biochemical diagnostic workup

Table 1.

**Table 1:** Typical biochemical findings in Sanjad-Sakati syndrome.

Parameter	Typical findings in SSS
Serum Calcium	↓ Decreased
Serum Phosphate	↑ Elevated
PTH Level	↓ Inappropriately low or undetectable
Serum Magnesium	Normal or low
Alkaline Phosphatase	Normal or slightly increased
Vitamin D (25-OH, 1,25-OH2)	Normal
Renal Function Tests	Normal initially
Blood Gas Analysis	Normal or mild metabolic alkalosis

### 5.3 Clinical systemic evaluation

Once hypoparathyroidism and dysmorphic features are confirmed, a comprehensive assessment for multisystem involvement should be performed (Table 2).

**Table 2:** Systemic manifestations and diagnostic tools in Sanjad-Sakati syndrome.

System	Findings	Diagnostic tool
Neurologic	Seizures, psychomotor/developmental delay,	EEG, Brain MRI
Respiratory	Recurrent infections, airway anomalies.	Chest x-ray, CT, and bronchoscopy
Renal	Nephrocalcinosis	Renal ultrasound
Dental	Hypodontia, enamel hypoplasia	Dental evaluation, panoramic x-rays
Ophthalmologic	Microphthalmia, optic nerve hypoplasia, corneal opacities	Fundoscopy
ENT	High-pitched voice, choanal stenosis	Nasoendoscopy
Bone	Delayed bone maturation, basal ganglia calcifications	Skull & Bone x-ray/CT

### 5.4 Genetic evaluation

Molecular testing for pathogenic variants in the TBCE gene located on chromosome 1q42–43 should be performed to confirm the diagnosis of SSS. The most common pathogenic variant identified in Middle Eastern populations is a 12–base pair deletion (c.155_166del; p.Ser52_Gly55del). Genetic counseling should be offered to parents and siblings to discuss inheritance patterns, recurrence risk, and options for carrier or prenatal testing.

### 5.5 Differential diagnosis exclusion

Table 3.

**Table 3:** Differential diagnoses of Sanjad-Sakati syndrome and key differentiating features.

Condition	Key differentiating features
DiGeorge syndrome (22q11.2 deletion syndrome)	▪ Cardiac defects (conotruncal anomalies; Tetralogy of Fallot, interrupted aortic arch). ▪ Thymic hypoplasia leading to T-cell immunodeficiency (not seen SSS). ▪ Characteristic facies: hooded eyelids, bulbous nasal tip, small ears with notched pinnae. ▪ Cleft palate/velopharyngeal insufficiency is common. ▪ Hypocalcaemia is often transient in infancy but may recur or persist because of hypoparathyroidism. ▪ Diagnosed via 22q11.2 microdeletion testing or chromosomal microarray, absent in SSS.
Kenny–Caffey syndrome type 1/TBCE-related recessive phenotype (historically distinguished from SSS)	▪ Shares some overlapping findings with SSS: growth retardation, hypocalcemia, and facial dysmorphism. ▪ Bone abnormalities are prominent: cortical thickening, medullary stenosis of long bones (not seen in SSS). ▪ Intellectual disability is usually milder. ▪ Both are caused by TBCE mutations, but SSS shows a more severe phenotype with global developmental impairment, more profound hypocalcemia, and typical Middle Eastern founder mutations.
Kenny–Caffey syndrome type 2 (FAM111A-related skeletal dysplasia)	▪ Shares overlap with SSS: short stature, hypocalcaemia due to hypoparathyroidism, and facial dysmorphism. ▪ Bone abnormalities are characteristic: cortical thickening and medullary stenosis of long bones, sometimes with delayed closure of a large fontanelle (not seen in SSS). ▪ Development and cognition are usually normal, unlike the typical global developmental impairment in SSS. ▪ Caused by heterozygous pathogenic FAM111A variants, often *de novo*, rather than biallelic TBCE variants in SSS.
Pseudohypoparathyroidism (PHP)	▪ Elevated PTH levels with end-organ resistance (SSS has low or inappropriately normal PTH) ▪ Albright hereditary osteodystrophy (AHO) phenotype in some subtypes: round face, short stature, brachydactyly, obesity—not characteristic of SSS. ▪ Serum phosphate is typically high, but PTH remains elevated, unlike SSS. ▪ Genetic testing reveals GNAS mutations.
Isolated congenital hypoparathyroidism	▪ Presents with hypocalcemia and low PTH without systemic dysmorphism or syndromic features. ▪ Normal growth and normal intelligence, unlike SSS. ▪ No characteristic craniofacial anomalies. ▪ May be due to mutations in PTH, GCM2, or CaSR.
Hypomagnesemia-induced hypocalcemia	▪ Hypocalcemia occurs secondary to severe hypomagnesemia, which suppresses PTH secretion. ▪ Reversible: calcium levels improve rapidly after magnesium repletion, unlike the persistent hypocalcemia in SSS. ▪ No dysmorphic features, growth failure, or global developmental delay. ▪ Causes may include malabsorption, medications (PPIs, diuretics), or renal Mg wasting.

### 5.6 Post-diagnosis recommendations

In SSS, the multisystem nature and chronic course of the disease necessitate continuous preventive management through a multidisciplinary team comprising pediatric endocrinologists, neurologists, nephrologists, ophthalmologists, dentists, and otolaryngologists. Lifelong calcium and vitamin D supplementation to maintain therapeutic levels remains the cornerstone of management, accompanied by regular monitoring of serum calcium, phosphate, magnesium, and PTH concentrations. This monitoring guides dose adjustment and helps prevent hypercalciuria and nephrocalcinosis, which may result from overtreatment and can be detected early through periodic renal ultrasonography.

**Figure 1:**
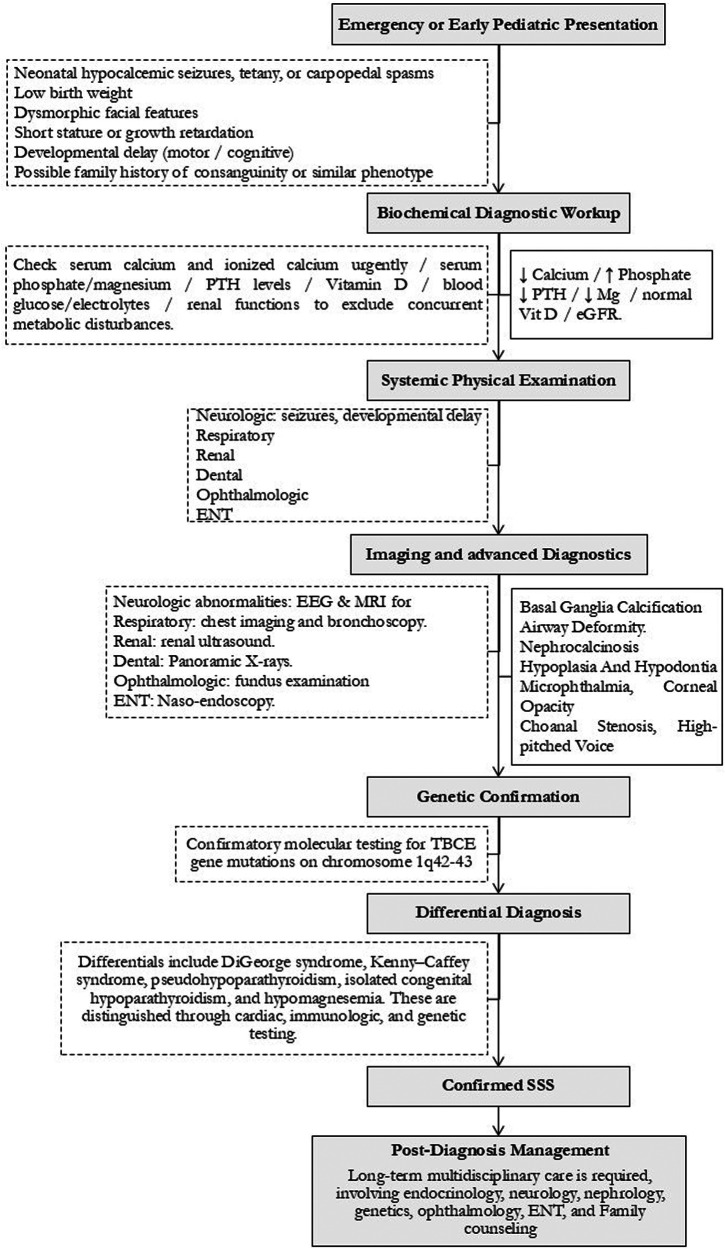
Diagnostic algorithm for Sanjad–Sakati syndrome (SSS).

Neurodevelopmental care aimed at mitigating cognitive delay should primarily rely on scheduled physiotherapy and speech therapy. Chest physiotherapy and routine follow-up by otolaryngology and pulmonology specialists are essential for patients with airway anomalies to prevent recurrent respiratory infections. Early dental evaluation during childhood—focusing on enamel hypoplasia, malocclusion, and delayed eruption—is crucial for anticipating complications that may impair speech or feeding. Ophthalmologic assessments are equally important to identify microphthalmia, optic nerve hypoplasia, or refractive errors requiring corrective management. Alongside these preventive measures, families should receive written emergency instructions detailing the recognition and management of hypocalcemic crises. This guidance should include early symptom identification and clear steps for administering oral or intravenous calcium as indicated (Figure 1).

## 6 Treatment

Management of SSS–related end-organ complications is multifaceted, requiring both acute intervention during emergencies and lifelong maintenance therapy to control hypocalcemic crises, minimize end-organ damage, and prevent systemic complications.

### 6.1 Emergency treatment

**Figure 2:**
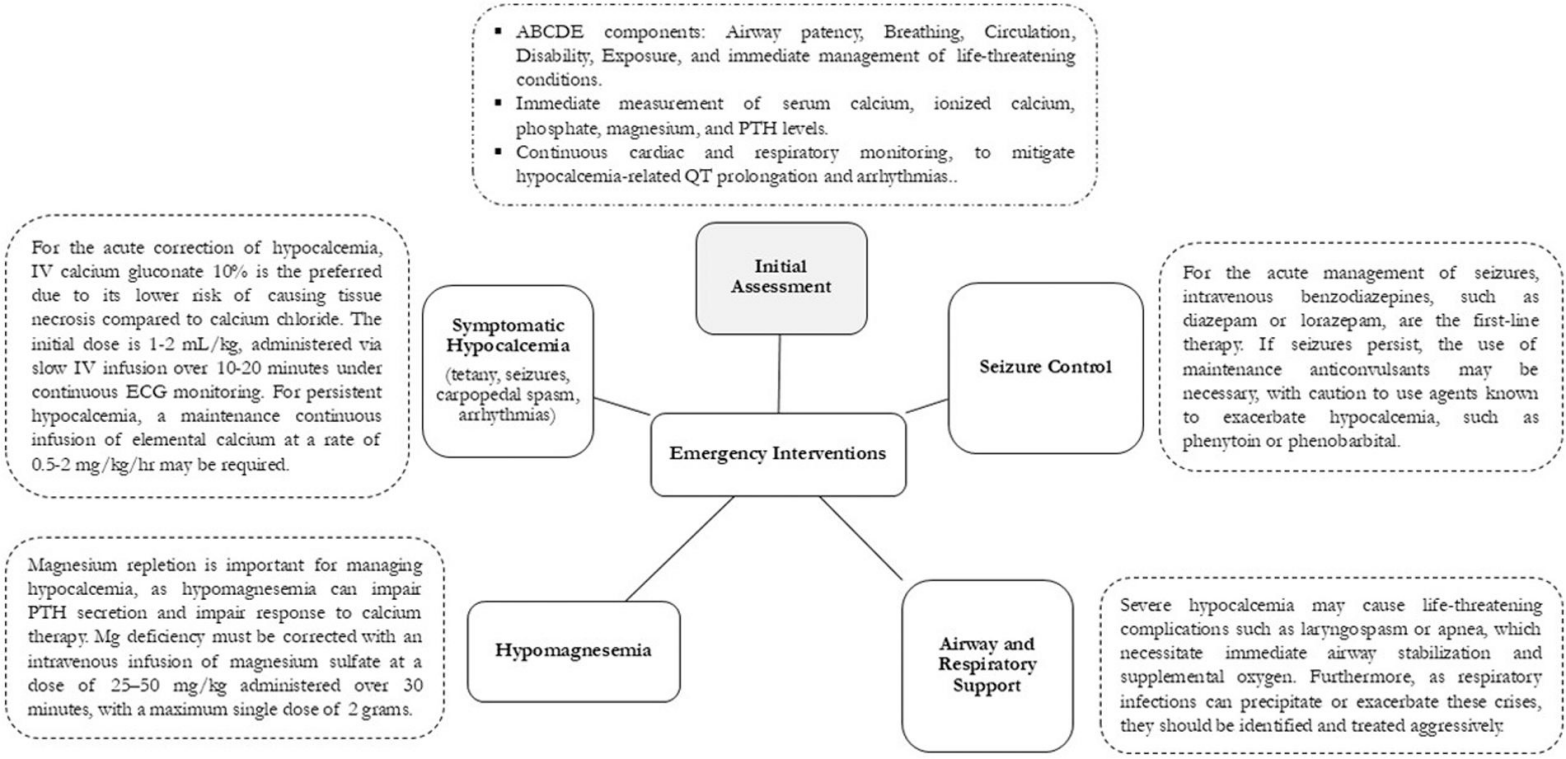
Emergency treatment of SSS manifestations.

Acute management of SSS-related crises focuses on the rapid correction of hypocalcemia and prompt control of neuromuscular symptoms to prevent life-threatening complications such as seizures and laryngospasm. In the largest longitudinal cohort of patients with hypoparathyroidism–retardation–dysmorphism syndrome, the leading causes of hospital admission were hypocalcemia (45%), fever (33%), seizures (25%), dehydration (13%), and hypoglycemia (12%). These findings support a broad emergency assessment that includes prompt metabolic correction, seizure management, blood glucose measurement, hydration assessment, and early evaluation for infection and respiratory compromise (47) (Figure 2).

#### 6.1.1 Airway management in SSS

Airway management in SSS is challenging due to characteristic craniofacial anomalies that cause upper-airway narrowing and reduced respiratory reserve. The biochemical disturbances act synergistically with these structural abnormalities, predisposing affected individuals to a difficult mask seal, challenging laryngoscopy and intubation, and restrictive pulmonary defects related to chest wall hypoplasia and recurrent lower-respiratory infections. These complex airway features underscore the need for a well-planned, multidisciplinary approach to airway management (41, 48). Khalil and colleagues reported significant difficulty securing the airway in a child with SSS, necessitating the use of a video laryngoscope, external laryngeal manipulation, and nasal intubation with Magill forceps to facilitate tube placement. Their report emphasized the importance of having a difficult-airway cart readily available and of anticipating the need for advanced intubation techniques (49). Similarly, Alshoaiby et al. described comparable airway challenges in a tracheostomized SSS patient, which mitigated upper-airway obstruction but highlighted the importance of securing the airway in a controlled environment and anticipating perioperative ventilatory complications (50). In a 12-year-old patient with SSS, Platis and colleagues identified midfacial hypoplasia, tonsillar hypertrophy, and restrictive pulmonary physiology as critical warning signs necessitating heightened vigilance during anesthesia and airway manipulation (48). Airway management in SSS should be undertaken in a well-equipped operating room with full anesthesia support and immediate access to a pediatric otolaryngologist or an experienced ENT specialist. Whenever possible, non-invasive ventilation techniques should be prioritized to minimize complications associated with repeated intubation attempts (51). When intubation is unavoidable, preparation for a difficult airway—including availability of video laryngoscopy with fiber-optic capability, endotracheal tubes of multiple sizes, and a skilled multidisciplinary team—is essential to maximize safety and optimize outcomes.

#### 6.1.2 Intravenous access considerations in SSS

Securing reliable intravenous (IV) access in children with SSS during episodes of shock or seizures can be extremely challenging and often meets the criteria for difficult intravenous access (DIVA). DIVA is defined as a situation in which multiple attempts or specialized techniques are required to establish and maintain patent IV access, most commonly due to small patient extremities, poor venous visibility or palpability, dehydration, and complex comorbidities (52). When IV access is urgently required, it is advisable for experienced personnel to attempt multiple peripheral IV insertions simultaneously rather than performing repeated sequential trials. According to the 2020 American Heart Association Pediatric Advanced Life Support guidelines, if peripheral access cannot be established within 60–90 s, intraosseous (IO) access should be initiated immediately (53).

The skeletal anomalies and reduced bone size characteristic of SSS may complicate conventional IO approaches. In resource-limited settings, expert opinion and case reports have described the use of an 18-gauge hypodermic needle as a last-resort IO cannula in critically ill pediatric patients when standard IO equipment is unavailable (54, 55). Although potentially lifesaving in emergencies, this method requires skilled healthcare providers and vigilant monitoring to prevent complications.

In addition to early IO planning, a stepwise escalation protocol for vascular access should include ultrasound-guided peripheral IV insertion performed by trained practitioners to facilitate cannulation of small or poorly visible veins (56). For patients who require frequent infusions, fluid resuscitation, or electrolyte correction, early consideration of central venous access is crucial when peripheral attempts repeatedly fail. Continuous provider training and accessibility of appropriate equipment—such as long flexible IV catheters, bedside ultrasonography, and sterile central-line kits—are essential to minimize procedural delays and reduce complication rates.

#### 6.1.3 Fever management in SSS

The immunodeficiency associated with SSS necessitates vigilant management of fever in affected children. Reported fatalities in SSS have often resulted from overwhelming bacterial sepsis, particularly due to Streptococcus pneumoniae and other encapsulated organisms. This reflects an underlying immune compromise that warrants prompt evaluation and empiric initiation of broad-spectrum antibiotic therapy. Antimicrobial treatment should be continued until blood culture results are negative and the patient demonstrates sustained clinical stability (57).

A recent immune-phenotyping study by David and colleagues involving nine children with SSS evaluated their functional immune responses and revealed several abnormalities. These included elevated total IgA and IgE levels, unexpectedly low anti-pneumococcal antibody titers despite prior immunization, reduced B-cell frequency, and a relative expansion of CD21lowCD27- B cells—a phenotype associated with impaired B-cell function. Additionally, reduced terminally differentiated CD8^+^ T cells, inverted CD4:CD8 ratios, and markedly impaired lymphocyte proliferation were observed. Despite prophylactic antibiotic use, the cohort exhibited considerable infectious morbidity: three patients developed bacteremia, and three contracted COVID-19, two of whom died (58). Based on the current literature, some patients with SSS may exhibit varying degrees of immunologic dysfunction; however, the extent and clinical relevance remain incompletely defined. Accordingly, management should emphasize vigilance, rapid assessment, and avoidance of delays in the initiation of empiric broad-spectrum antibiotics when clinically indicated. Preventive measures—including vaccination, appropriate use of prophylactic antibiotics, and regular follow-up with immunology specialists—are critical given the potential for immune dysregulation in this population.

#### 6.1.4 Constipation

Constipation is a common gastrointestinal complication in patients with SSS, primarily resulting from metabolic disturbances and, in some cases, visceral myopathy affecting the intestinal smooth muscle. These pathophysiologic factors predispose patients to severe constipation that may progress to acute, life-threatening intestinal pseudo-obstruction. If unrecognized or untreated, this condition can lead to progressive colonic dilation, intestinal failure, sepsis, and early mortality (59).

Initial management involves early abdominal imaging to identify pseudo-obstruction, followed by prompt initiation of oral or rectal laxatives, enemas, or glycerin suppositories to relieve fecal loading and prevent progressive colonic distension. Supportive care measures, including intravenous hydration and correction of hypocalcemia and hypokalemia, are essential because electrolyte disturbances can further impair gastrointestinal motility.

### 6.2 Long-term treatment and monitoring

The long-term management of SSS requires a comprehensive, multidisciplinary approach aimed at maintaining biochemical stability, promoting optimal physical growth and neurodevelopment, and minimizing complications associated with chronic hypocalcemia and endocrine dysfunction.

#### 6.2.1 Calcium and vitamin D supplementation

Patients with SSS have congenital hypoparathyroidism, which results in persistent hypocalcemia and hyperphosphatemia. The cornerstone of long-term management is lifelong oral supplementation with elemental calcium (50–75 mg/kg/day) in combination with active vitamin D analogues (0.25–1 μg/day), such as calcitriol or alfacalcidol, to maintain serum calcium concentrations within the target range (60–62). Regular monitoring of serum calcium, phosphate, magnesium, alkaline phosphatase, and urinary calcium levels is essential to ensure biochemical control and to prevent nephrocalcinosis or renal impairment.

#### 6.2.2 Magnesium repletion

Magnesium homeostasis plays a vital role in calcium metabolism and PTH function. The coexistence of hypomagnesemia (<0.65 mmol/L) and hypocalcemia in patients with SSS can exacerbate neuromuscular irritability and precipitate seizures, even when calcium and vitamin D supplementation are adequate. Magnesium deficiency impairs both PTH secretion and peripheral responsiveness to PTH, thereby aggravating the biochemical abnormalities associated with congenital hypoparathyroidism. Oral magnesium supplementation, typically in the form of magnesium oxide (50–100 mg/kg/day), is recommended for patients with low or borderline serum magnesium concentrations. The target serum magnesium range for maintenance therapy is 0.8–1.0 mmol/L (2.0–2.4 mg/dL). During the initial phase of repletion, serum magnesium should be monitored weekly, and once stable levels are achieved, monitoring may be performed monthly or quarterly (63, 64).

#### 6.2.3 Hyperphosphatemia management

Hyperphosphatemia is a common biochemical abnormality in patients with SSS, primarily resulting from PTH deficiency, which impairs renal phosphate excretion. Persistent hyperphosphatemia exacerbates hypocalcemia by forming insoluble calcium–phosphate complexes, promotes soft-tissue and vascular calcification, and contributes to renal complications such as nephrocalcinosis. Therefore, controlling phosphate levels represents a cornerstone of long-term management in SSS (65).

Patients should avoid foods rich in inorganic phosphate, including dairy products, processed meats, carbonated beverages, and foods containing phosphate additives. Low-phosphate formulas may be used under medical supervision when appropriate (66). When dietary restriction alone fails to maintain phosphate within target limits, phosphate-binding agents are indicated to reduce intestinal phosphate absorption. Calcium-based phosphate binders, such as calcium carbonate or calcium acetate, provide a dual benefit—controlling hyperphosphatemia while correcting hypocalcemia. Non–calcium-based binders, such as sevelamer hydrochloride, are recommended for patients with normal or elevated serum calcium levels. Sevelamer may be initiated at 400–800 mg per meal, with subsequent dose adjustment according to body weight and serum phosphate concentrations (67, 68).

Regular monitoring of phosphate metabolism should include serum calcium, phosphate, alkaline phosphatase, urinary calcium, and calculation of the calcium–phosphate product. The therapeutic targets are to maintain serum calcium within the low-normal range (8.0–8.5 mg/dL) and serum phosphate within the upper-normal to mildly elevated range (4.5–5.5 mg/dL). A critical goal is to keep the calcium–phosphate product below 55 mg^2^/dL^2^ to minimize the risk of ectopic soft-tissue calcification.

#### 6.2.4 Growth and nutritional support

Intrauterine growth restriction and postnatal short stature are common in patients with SSS. These manifestations result from a combination of congenital endocrine dysfunction—manifested as hypoparathyroidism with disturbances in calcium–phosphate homeostasis—together with intrinsic developmental defects related to microtubule dysfunction, feeding difficulties, and occasionally visceral or gastrointestinal dysmotility (7). Management of growth failure should be patient-centered and multidisciplinary. A comprehensive nutritional assessment at the time of diagnosis should include accurate anthropometric measurements (weight, length/height, head circumference, and weight-for-length *z*-scores), estimation of caloric requirements, evaluation of feeding and oral–motor function, and screening for nutrient and electrolyte deficiencies (2). Early involvement of a pediatric dietitian is essential to individualize dietary plans and minimize the energy deficits contributing to growth failure. Chronic hypocalcemia, hyperphosphatemia, and recurrent metabolic crises suppress appetite, increase catabolism, and impair bone and soft-tissue growth. Therefore, achieving stable serum calcium and vitamin D levels forms the foundation of nutritional rehabilitation, alongside correction of anemia and management of gastrointestinal disturbances or other conditions that reduce effective nutrient intake. GH therapy has been attempted in some cohorts of SSS patients to improve linear growth; however, several case reports have documented limited or absent growth response, suggesting partial GH resistance (23, 24).

#### 6.2.5 Management of systemic complications

Neurological complications secondary to chronic hypocalcemia or structural brain abnormalities in patients with SSS may include developmental delay, hypotonia, and seizures. Early correction of serum calcium and magnesium concentrations is crucial to prevent seizures and metabolic encephalopathy. Antiepileptic therapy should be individualized, avoiding agents that can exacerbate calcium metabolism disturbances, such as phenytoin or phenobarbital (69). Neuroimaging may be warranted to evaluate basal ganglia calcifications associated with persistent hypocalcemia.

Because long-term calcium and vitamin D supplementation increases the risk of nephrocalcinosis, nephrolithiasis, and renal insufficiency, management should aim to maintain serum calcium within the low-normal range and to prevent hypercalciuria through regular monitoring of urine calcium-to-creatinine ratios and renal ultrasonography every six months for early detection of nephrocalcinosis. In confirmed cases of nephrocalcinosis, reducing calcium and vitamin D intake, ensuring optimal hydration, and administering thiazide diuretics (hydrochlorothiazide 0.5–1 mg/kg/day) may help decrease urinary calcium excretion (70).

Prolonged hypocalcemia and vitamin D deficiency can contribute to rickets-like musculoskeletal deformities and impaired skeletal growth. In cases of severe growth failure, GH therapy may be considered; however, treatment outcomes remain uncertain. Orthopedic surgical intervention may also be required to address skeletal dysplasia, kyphosis, or limb deformities. Ophthalmologic complications such as microphthalmia, strabismus, and refractive errors require early assessment and correction with eyeglasses or surgical procedures as indicated. Likewise, dental abnormalities—including microdontia, delayed eruption, enamel hypoplasia, and malocclusion—necessitate regular dental follow-up and a preventive care plan to reduce infection risk and preserve oral and masticatory function.

Patients prone to recurrent respiratory infections, whether due to craniofacial dysmorphism, structural airway anomalies, or possible immunodeficiency, should follow a comprehensive prophylactic plan that includes airway clearance techniques, up-to-date vaccination against respiratory pathogens, and early administration of broad-spectrum antibiotics when indicated. Periodic cardiovascular evaluation is warranted, particularly in patients with prolonged QT intervals secondary to hypocalcemia or suspected structural cardiac defects. In addition, scheduled follow-up with pulmonology and immunology specialists ensures multidisciplinary continuity of care.

#### 6.2.6 Genetic testing and family counseling

Given the high rate of consanguinity in regions where SSS is prevalent and the established autosomal recessive inheritance pattern of the disease, comprehensive genetic counseling represents a vital component of long-term family care. Counseling sessions should provide families with clear information about recurrence risks, the significance of carrier testing among relatives, and the availability of premarital genetic screening. Furthermore, early identification of siblings carrying TBCE gene mutations through family-based screening is crucial, as it enables timely initiation of management and optimization of clinical outcomes.

#### 6.2.7 Psychosocial and educational support

SSS profoundly affects the psychosocial well-being and educational development of both affected children and their families. The psychosocial burden stems from intellectual disability, growth failure, facial dysmorphism, and recurrent medical emergencies, all of which contribute to social isolation, emotional distress, financial strain, and a diminished family quality of life. Early neurodevelopmental interventions—such as physical, occupational, and speech therapy—are essential to optimize developmental outcomes. Individualized education plans (IEPs) tailored to each child's cognitive profile and learning abilities should be designed collaboratively by healthcare professionals, educators, and families. These plans must be periodically reviewed and updated to align with evolving developmental goals.

Given the chronic and multisystemic nature of the disease, families often experience significant emotional and financial stress. Genetic counselors and psychosocial support specialists play a critical role in helping families understand emergency preparedness, reproductive and prenatal diagnostic options, and coping mechanisms. Participation in social support groups and community-based rehabilitation programs can further address caregiver challenges and enhance family resilience. For school-aged children, teachers and school health personnel should be educated about the child's condition, recognition of early hypocalcemic symptoms, and implementation of individualized emergency response protocols.

#### 6.2.8 Emergency prevention strategies

Because of the high risk of acute hypocalcemic crises, seizures, and life-threatening respiratory or metabolic complications resulting from poor treatment adherence, infections, or dehydration, emergency prevention in SSS remains a cornerstone of long-term management. Comprehensive preventive strategies should integrate metabolic control, caregiver education, infection prevention, and rapid response planning to minimize avoidable morbidity and mortality. Regular monitoring of serum calcium, phosphate, magnesium, and PTH concentrations every two weeks during the initial management phase—and quarterly once biochemical stability is achieved in older children—is essential for early detection of abnormalities before clinical deterioration occurs. Caregivers must receive clear, written instructions on dose adjustments, medication schedules, and the importance of treatment adherence, as lapses in therapy often precede emergency admissions.

Families should be trained to recognize early warning signs of hypocalcemia, such as perioral tingling, muscle cramps, carpopedal spasms, or seizures, and to seek immediate medical attention. Family-directed management handouts should include a list of the patient's medications, emergency contact numbers, and concise guidance for healthcare providers unfamiliar with SSS regarding its acute management. Additionally, caregivers should be advised to maintain adequate environmental humidity, avoid exposure to respiratory irritants, and monitor for symptoms of sleep-disordered breathing.

## 7 Prognosis

The prognosis of SSS primarily depends on the severity of hypocalcemia, In the largest longitudinal cohort, mortality was 52%, with pneumonia, septic shock, and meningitis accounting for most deaths, emphasizing the importance of early infection recognition and preventive care in this population. The timeliness and effectiveness of early biochemical correction, the extent of multiorgan involvement, and the adequacy of supportive care. SSS is generally associated with high infant and childhood mortality, mainly due to refractory seizures and recurrent respiratory tract infections. However, outcomes have improved considerably with early diagnosis, vigilant metabolic monitoring, and structured long-term pharmacologic management, resulting in greater metabolic stability and enhanced survival.

Despite these advancements, patients with SSS continue to experience significant long-term morbidity, including persistent neurodevelopmental delay, short stature, and varying degrees of intellectual disability that may not fully normalize even with optimal treatment (59). Life expectancy varies widely: while some patients survive into adulthood, many continue to experience moderate functional limitations and require lifelong multidisciplinary follow-up. Mortality in older children and adults is most often related to respiratory complications, severe infections, gastrointestinal abnormalities, or uncontrolled metabolic disturbances (47).

## 8 Conclusion

SSS is a complex pediatric genetic disorder characterized by the combined effects of congenital hypoparathyroidism, microtubule dysfunction, and multisystem dysmorphology. These interrelated abnormalities lead to recurrent hypocalcemic crises, respiratory and renal complications, and immune dysfunction.

This review underscores the importance of early recognition of the characteristic biochemical profile and dysmorphic features, along with prompt management of acute complications such as seizures, laryngospasm, respiratory failure, and sepsis. Effective emergency care relies primarily on timely correction of calcium and magnesium imbalances, meticulous airway management, and thorough evaluation of multisystem involvement Long-term stabilization requires a coordinated, multidisciplinary approach encompassing endocrine management, renal surveillance, neurodevelopmental support, nutritional planning, ophthalmologic and dental care, and proactive infection prevention.

Ultimately, the prognosis of affected children depends on prompt emergency stabilization, consistent metabolic and developmental follow-up within a multidisciplinary framework, and family-centered education and genetic counseling—particularly in populations with a high prevalence of consanguinity.
